# Supplementary material for: Transforming Growth Factor Beta 2 (TGFB2) mRNA Levels, in Conjunction with Interferon-Gamma Receptor Activation of Interferon Regulatory Factor 5 (IRF5) and Expression of CD276/B7-H3, Are Therapeutically Targetable Negative Prognostic Markers in Low-Grade Gliomas
Source: Cancers (Basel). 2024 Mar 19;16(6):1202. doi: 10.3390/cancers16061202 (PMC10968971; doi:10.3390/cancers16061202)
Supplement: Supplementary file 1 [file cancers-16-01202-s001.zip › cancers-2895478-supplementary.pdf]

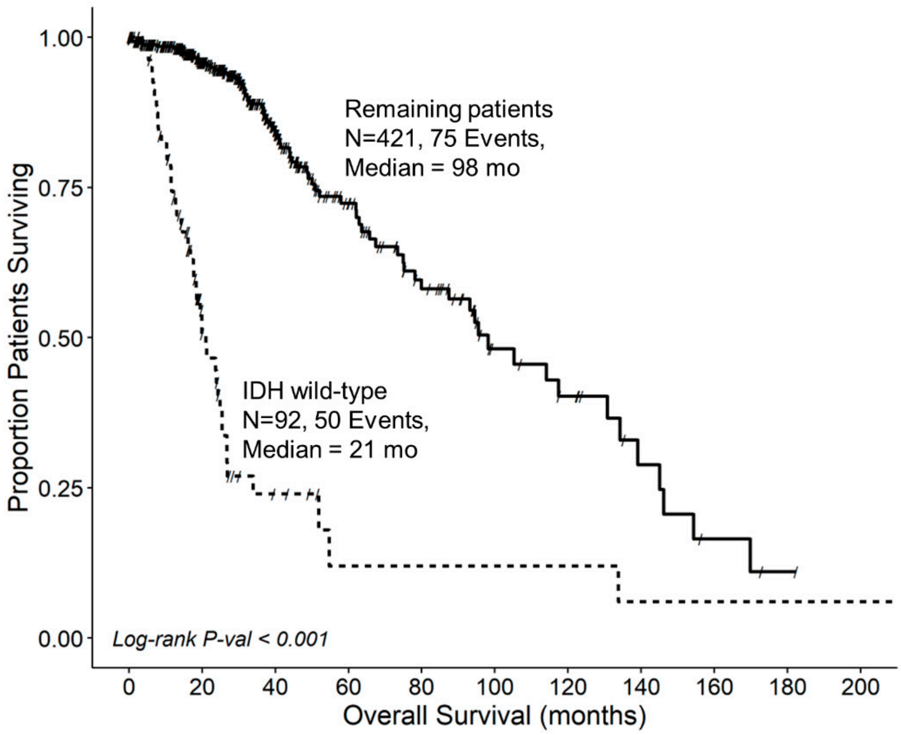

Figure S1. Prognostic impact of IDHwt genotype in LGG patients. We analyzed clinical metadata and RNA sequencing data from 513 patients who were diagnosed with LGG (https://www.cbioportal.org/study/summary?id=lgg_tcga_pan_can_atlas_2018). Our goal was to determine the correlation between IDH mutational status and overall survival time by comparing 92 IDHwt LGG patients with the remaining 421 patients. The median OS time for the IDHwt group was 21 months (95% CI: 17.7 - 25.5 months; N=92; 50 events). Of the 421 remaining patients, they experienced a significant increase in the median OS time of 98 months (95% CI: 80 – 134 months; 75 events; Log-rank Chi-square = 63.3, P<0.0001). There was a significant over-representation of patients with high TGFB2 and IFNGR2 mRNA expression in the IDHwt group of patients compared to the remaining patients: whereby 51 out of 421 remaining patients (12%) had high TGFB2 expression, while 78 out of 92 IDHwt patients (85%) had high TGFB2 expression (Odds ratio = 40, Fishers Exact Test, P<0.0001)); and 64 out of 421 remaining patients (15%) had high IFNGR2 expression, while 65 out of 92 IDHwt patients (71%) had high IFNGR2 expression (Odds ratio = 13.3, Fishers Exact Test, P<0.0001).

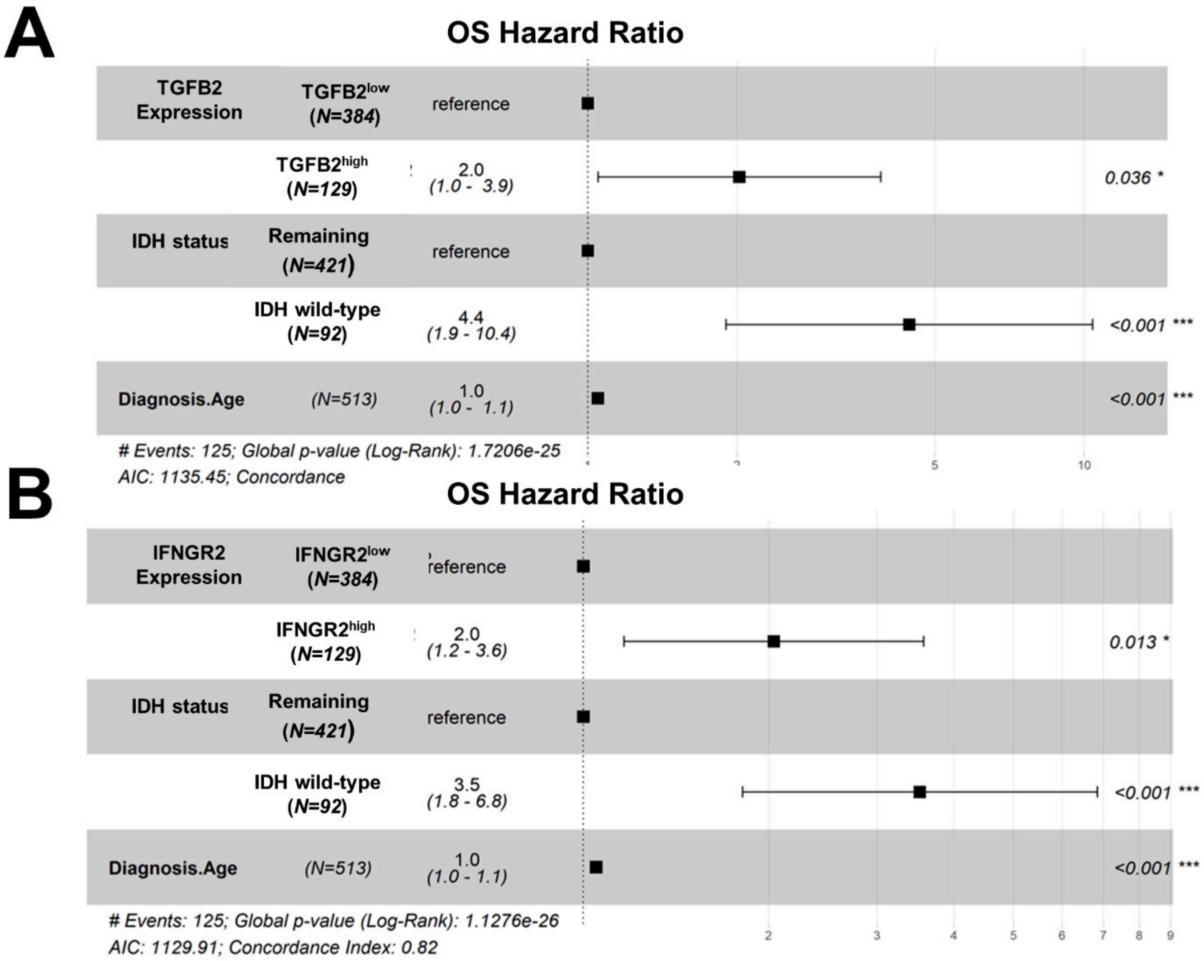

Figure S2. Prognostic impact of IDHwt genotype and TGFB2 or IFNGR2 mRNA expression in LGG patients using a multivariate Cox proportional hazards model with an interaction term. To evaluate the independent effects of TGFB2 and IDHwt mutational status on OS, multivariate analyses were carried out utilizing the Cox proportional hazards model. This analysis controlled for age at diagnosis. The model included (i) The mRNA expression level for TGFB2 (A) or IFNGR2 (B) as a categorical variable comparing high versus low mRNA expression levels at 25% cut-off for expression values, (ii) IDHwt versus remaining patients; (iii) age at diagnosis; and, (iv). An interaction term for mRNA expression levels x IDH mutational status. [**A**] The HR for patients in the TGFB2^{high} group showed a significant increase, with an HR (95% CI range) of 2.02 (1.05-3.89) (P = 0.036 compared to the remaining reference group of patients). Similarly, patients in the IDHwt group also showed a significant increase in HR, with an HR (95% CI range) of 4.44 (1.9-10.4) (P < 0.001 versus the remaining patients). Age at diagnosis was found to be significantly associated with an increase in HR, with an HR (95% CI range) of 1.05 (1.03-1.06) and a P value of less than 0.001. There was not a significant interaction between the TGFB2 mRNA expression level and IDH mutational status (HR (95% CI range) of 0.63 (0.21-1.88) and a P value of 0.41). [**B**] In analyzing the interaction between IFNGR2 and IDHwt, it was found that patients in the IFNGR2^{high} group exhibited a significant increase in HR (HR (95% CI range) = 2.04 (1.16-3.57); P = 0.013). Similarly, patients in the IDHwt group also showed a significant increase in HR (HR (95% CI range) = 3.52 (1.81-6.84); P < 0.001), controlling for the age at diagnosis (HR (95% CI range) = 1.05 (1.03-1.07); P < 0.001) and the interaction term (HR (95% CI range) = 0.96 (0.4-2.31); P = 0.927). * denotes P < 0.05, ** denoted P < 0.01, *** denotes P< 0.001.

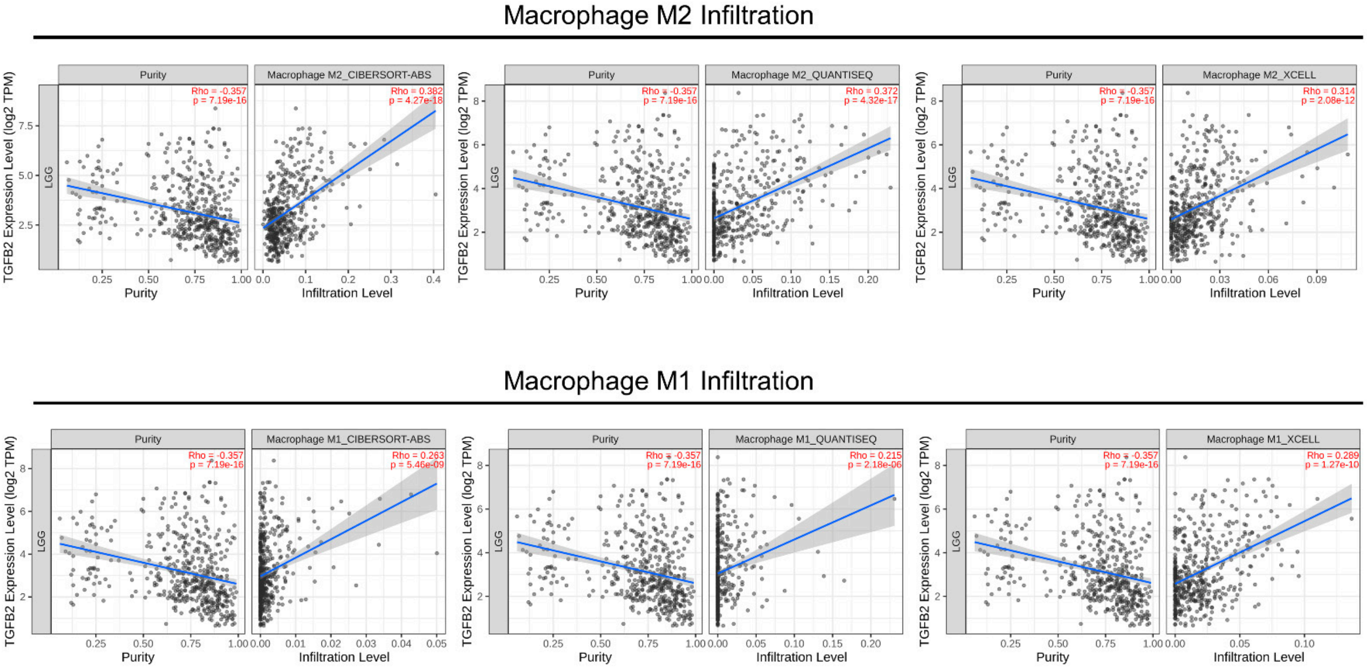

Figure S3. TGFB2 mRNA expression levels are positively correlated with Macrophage infiltration into LGG tumors. We estimated the correlation of TGFB2 and macrophage immune-cell infiltration in LGG tumors using the algorithms provided in the TIMER2.0 (http://timer.cistrome.org/) web tool compiled for the Cancer Genome Atlas (TCGA). Depicted are the purity-adjusted Spearman's rho correlations utilizing CIBERSORT-ABS, QUANTISEQ, and XCELL immune deconvolution methods to estimate infiltration of M2 and M1 macrophages in LGG tumors. TGFB2 expression values (RSEM estimated TPM values) show significant correlations with both M1 and M2 macrophages using the 3 deconvolution methods.

Table S1. Comparing the levels of mRNA expression in normal brain tissue and tumor samples obtained from LGG patients.

	Normal tissue (N=1141)		Primary tumor (N=509)		FDR adjusted Linear Contrast	
Gene	Mean ± SEM	Median(Range)	Mean ± SEM	Median(Range)	Fold Change (Tumor/Normal)	P
CD276	3.13 ± 0.04	3.31 (-9.97 - 5.98)	5.15 ± 0.03	5.07 (3.12 - 7.86)	4.0	<0.0001
CD68	1.97 ± 0.06	2.07 (-9.97 - 8.59)	5.93 ± 0.06	5.97 (0.78 - 9.82)	15.6	<0.0001
CD86	-0.74 ± 0.06	-0.73 (-9.97 - 6.18)	2.41 ± 0.06	2.43 (-2.39 - 5.72)	8.9	<0.0001
IFNGR1	4.33 ± 0.04	4.28 (-9.97 - 7.47)	5.98 ± 0.03	5.98 (4.13 - 7.62)	3.1	<0.0001
IFNGR2	4.21 ± 0.04	4.31 (-9.97 - 6.78)	5.17 ± 0.03	5.03 (3.72 - 7.23)	1.9	<0.0001
IRF1	2.08 ± 0.04	1.95 (-9.97 - 9.28)	3.16 ± 0.05	2.93 (0.99 - 7.09)	2.1	<0.0001
IRF5	1.1 ± 0.04	1.22 (-9.97 - 5.01)	2.73 ± 0.05	2.77 (-2.18 - 5.2)	3.1	<0.0001
MSR1	-0.27 ± 0.06	-0.39 (-9.97 - 7.07)	2.3 ± 0.07	2.23 (-2.11 - 6.96)	6.0	<0.0001
STAT1	3.63 ± 0.04	3.74 (-9.97 - 8.41)	5.17 ± 0.04	5.03 (2.55 - 8.81)	2.9	<0.0001
TGFB1	2.45 ± 0.04	2.43 (-9.97 - 6.11)	4.5 ± 0.04	4.64 (2.17 - 6.7)	4.1	<0.0001
TGFB2	1.48 ± 0.04	1.67 (-9.97 - 5.08)	2.64 ± 0.08	2.43 (-2.31 - 8.3)	2.2	<0.0001
TGFB3	3.73 ± 0.04	3.58 (-9.97 - 7.87)	3.85 ± 0.03	3.84 (0.93 - 6.15)	1.1	0.1

Table S2. Impact of mRNA levels on the OS of LGG patients using Univariate Cox Proportional Hazards models. Expression levels for each gene were ranked according to log2 TPM values for 513 LGG patients and stratified into two groups for high expression (greater than or equal to 25th percentile) and low expression (lower 75th percentile). A univariate Cox regression model was utilized to determine hazard ratios and (95% confidence intervals) comparing high versus low expression for each of the treatments.

	All LGG Patients (N=513)		Temozolomide treated (N=259)		Radiation treated (N=299)		Bevacizumab treated (N=48)		Lomustine treated (N=39)	
Gene	Hazard ratio (95% CI)	P	Hazard ratio (95% CI)	P	Hazard ratio (95% CI)	P	Hazard ratio (95% CI)	P	Hazard ratio (95% CI)	P
CD276	2.69 (1.88-3.85)	P < 0.001	2.26 (1.46-3.5)	P < 0.001	2.31 (1.54-3.47)	P < 0.001	2.59 (1.22-5.49)	0.013	8.37 (2.51-27.98)	0.001
CD68	1.72 (1.19-2.49)	0.004	1.49 (0.93-2.39)	0.099	1.57 (1.03-2.37)	0.035	2.07 (0.99-4.36)	0.055	4.62 (1.59-13.41)	0.005
CD86	1.83 (1.26-2.66)	0.002	1.21 (0.76-1.94)	0.423	1.34 (0.87-2.05)	0.182	1.71 (0.81-3.6)	0.158	4.43 (1.51-13)	0.007
IFNGR1	1.04 (0.7-1.53)	0.861	0.8 (0.5-1.29)	0.366	0.79 (0.51-1.22)	0.283	1.17 (0.56-2.44)	0.673	1.19 (0.51-2.8)	0.686
IFNGR2	3.32 (2.33-4.73)	P < 0.001	3.02 (1.92-4.75)	P < 0.001	2.34 (1.56-3.5)	P < 0.001	3.84 (1.67-8.81)	0.002	5.76 (1.98-16.79)	0.001
IRF1	2.67 (1.85-3.86)	P < 0.001	1.83 (1.18-2.83)	0.007	1.96 (1.28-2.99)	0.002	1.58 (0.78-3.21)	0.206	2.97 (1.24-7.12)	0.014
IRF5	1.83 (1.26-2.66)	0.002	1.45 (0.92-2.29)	0.107	1.59 (1.05-2.41)	0.03	1.17 (0.58-2.35)	0.658	2.11 (0.91-4.87)	0.081
MSR1	3 (2.1-4.28)	P < 0.001	2.54 (1.62-3.97)	P < 0.001	2.26 (1.51-3.39)	P < 0.001	2.74 (1.25-5.99)	0.012	5.8 (1.96-17.16)	0.002
STAT1	2.52 (1.76-3.6)	P < 0.001	1.86 (1.2-2.87)	0.006	1.73 (1.15-2.6)	0.009	1.32 (0.65-2.66)	0.439	1.49 (0.58-3.79)	0.403
TGFB1	1.5 (1.03-2.19)	0.033	1.13 (0.71-1.78)	0.609	1.15 (0.75-1.76)	0.535	0.94 (0.47-1.89)	0.866	2.13 (0.92-4.92)	0.077
TGFB2	3.92 (2.74-5.61)	P < 0.001	4.01 (2.61-6.17)	P < 0.001	3.56 (2.38-5.34)	P < 0.001	4.42 (2.1-9.29)	P < 0.001	7.47 (2.29-24.35)	0.001
TGFB3	1.91 (1.31-2.77)	0.001	2.15 (1.38-3.35)	0.001	2 (1.32-3.04)	0.001	1.57 (0.76-3.28)	0.225	2.77 (1.02-7.52)	0.045
